# Association of Circulating Tumor DNA With Disease-Free Survival in Breast Cancer: A Systematic Review and Meta-analysis

**DOI:** 10.1001/jamanetworkopen.2020.26921

**Published:** 2020-11-19

**Authors:** Carolyn Cullinane, Christina Fleming, Donal Peter O’Leary, Fara Hassan, Louise Kelly, Martin J. O’Sullivan, Mark Antony Corrigan, Henry Paul Redmond

**Affiliations:** 1 Department of General and Breast Surgery, Cork University Hospital, Cork, Ireland; 2 Cork Breast Research Centre, University College Cork, Cork, Ireland

## Abstract

**Importance:**

Fragmented DNA is continuously released into the circulation following apoptosis and necrosis of both cancerous and noncancerous cells; when it is released by cancer cells, it is specifically known as circulating tumor DNA (ctDNA). Previous studies have suggested that ctDNA can reflect tumor burden and guide potential therapeutic targets.

**Objective:**

To determine the association of ctDNA with breast cancer disease-free survival (DFS) and progression-free survival in early, locally advanced, and metastatic breast cancer.

**Data Sources:**

An electronic search was conducted using the Cochrane Library, ScienceDirect, PubMed, and Embase from July 30, 2019, to October 31, 2019; all languages were included. The following search terms were used: ctDNA OR circulating tumor DNA OR liquid biopsy AND breast cancer OR breast carcinoma OR breast tumor AND prognosis OR survival. All titles were screened, and the appropriate abstracts were reviewed. If any data were missing, the authors contacted the study authors for permission to access data and extrapolate hazard ratios (HRs).

**Study Selection:**

To be included in the analysis, the studies had to meet the following prespecified inclusion criteria: (1) a ctDNA blood sample was measured; (2) DFS, progression-free survival, or relapse-free survival was reported as an HR; and (3) the patient population only had breast cancer. Retrospective and prospective observational cohort studies were included.

**Data Extraction and Synthesis:**

Two authors (C.C. and C.F.) independently reviewed the literature. All data were recorded independently by both authors and were compared at the end of the reviewing process to limit selection bias. Duplicates were removed and any disparities were clarified. Data were pooled using a fixed-effects or random-effects model according to the study heterogeneity. This study adhered to the guidelines of the Preferred Reporting Items for Systematic Reviews and Meta-Analysis (PRISMA) and Meta-Analysis of Observational Studies in Epidemiology (MOOSE).

**Main Outcomes and Measures:**

The primary outcome was the association of ctDNA with DFS or relapse-free survival in breast cancer. Secondary outcomes focused on subgroup analysis in the setting of early breast cancer and metastatic breast cancer.

**Results:**

From a total of 263 publications found using the predefined search terms, data from 8 studies (3.0%) reporting on 739 patients in total were suitable for inclusion. Circulating tumor DNA gene variation detection (both before and after treatment) was statistically significantly associated with shorter DFS (HR, 4.44; 95% CI, 2.29-8.61; *P* < .001). Detection of ctDNA was statistically significantly associated with a reduction in DFS in both the early breast cancer subgroup (HR, 8.32; 95% CI, 3.01-22.99; *P* < .001) and the metastatic or locally advanced subgroup (HR, 1.91; 95% CI, 1.35-2.71; *P* < .001). Pretreatment and posttreatment plasma sample collection was analyzed in both early and metastatic groups. The posttreatment group encompassed both surgical and oncologic therapy. Pretreatment plasma detection of ctDNA was statistically significantly associated with reduced DFS (HR, 3.30; 95% CI, 1.98-5.52; *P* < .001). Posttreatment sampling of ctDNA failed to achieve statistical significance (HR, 8.17; 95% CI, 1.01-65.89; *P* = .05).

**Conclusions and Relevance:**

In this systematic review and meta-analysis, elevated plasma ctDNA was associated with a high risk of relapse. This finding suggests that plasma ctDNA may provide an excellent method to stratify risk and personalize patient follow-up.

## Key Points

**Question:**

Is circulating tumor DNA (ctDNA) detection associated with unfavorable breast cancer outcomes?

**Findings:**

This systematic review and meta-analysis of 8 studies comprising 739 patients found that elevated ctDNA levels were associated with poorer cancer outcomes in patients with early, locally advanced, and metastatic disease; ctDNA mutation detection (both before and after treatment) was statistically significantly associated with shorter disease-free survival and a reduction in disease-free survival in both the early breast cancer subgroup and the metastatic and locally advanced subgroup.

**Meaning:**

These findings suggest that monitoring ctDNA levels in breast cancer has the potential to gauge response to treatment and aid in early detection of disease progression or recurrence.

## Teaser

This systematic review and meta-analysis uses data from studies retrieved from a Cochrane Library, ScienceDirect, PubMed, and Embase search to investigate the association of circulating tumor DNA with disease-free survival in patients with breast cancer.

## Introduction

Breast cancer is the most common cancer affecting women worldwide.^1^ Despite significant advances in treatment regimes, screening, and surveillance, the risk of disease recurrence persists for years after the initial diagnosis.^2^ Local and systemic breast cancer recurrence can be difficult to detect early owing to the lack of associated clinical symptoms and the limitations of radiologic testing. Therefore, there is considerable interest in new, noninvasive methods for early disease detection.

Liquid biopsy is a noninvasive technique that can yield important diagnostic, therapeutic, and prognostic information in many types of cancer.^3^ A component of liquid biopsy known as circulating tumor DNA (ctDNA) has shown promise in the detection of breast cancer and clinical and cancer outcomes.^4,5^ Circulating tumor DNA is found within the blood of patients with cancer owing to the continuous release of fragmented DNA from necrotic and apoptotic cancer cells.^6^ Circulating tumor DNA is a subgroup of cell-free DNA that is present in healthy individuals and is released from nonmalignant cells,^7^ and it accounts for between 0.01% and 50% of the total cell-free DNA in patients with cancer.^8^ Circulating tumor DNA levels are influenced by tumor size and burden; therefore, serial measurement of ctDNA can provide real-time noninvasive monitoring of response to treatment and early detection of recurrence.^9^

Personalized medicine and precision oncology emerged as an exciting concept in the late 1990s with the advent of trastuzumab for *ERBB2* (*HER2/neu*)–positive breast tumors and imatinib for *BCR-ABL* chronic myeloid leukemia.^10^ The key objective of personalized therapy is to provide the most accurate and effective oncologic treatment to patients based on tumor and individual profiling.^7^ Circulating tumor DNA can be used not only as a blood-based biomarker to assess response to treatment but also to potentially identify key treatment targets.^11^ In breast cancer, *PIK3CA* and *BRCA1* are common gene variations, and significant research has been invested in targeting them. Examples of these treatments include the poly adenosine diphosphate ribose polymerase inhibitor olaparib, which is more effective in the presence of *BRCA1*, whereas the presence of *PIK3CA* would suggest sensitivity to the mammalian target of rapamycin (mTOR) inhibitor everolimus.^12,13^

Monitoring ctDNA levels in breast cancer has the potential to gauge response to treatment and aid in early detection of disease progression or recurrence. Thus, the aim of this study was to determine the association of ctDNA with breast cancer disease-free survival (DFS) and progression-free survival in early, locally advanced, and metastatic breast cancer.

## Methods

This study adhered to the Preferred Reporting Items for Systematic Reviews and Meta-analyses (PRISMA) and Meta-analysis of Observational Studies in Epidemiology (MOOSE) reporting guidelines.^14^ Analysis and results were extracted from previous ethically approved studies; therefore, patient consent and ethical approval were not required as per the Clinical Research Ethics Committee of the Cork Teaching Hospitals. This study was prospectively registered on PROSPERO on September 24, 2019 (152173).^15^

### Search Strategy

An electronic search was conducted using the Cochrane Library, ScienceDirect, PubMed, and Embase. All studies were from July 30, 2019, to October 31, 2019, and all languages were included. The following search terms or Medical Subject Headings (MeSH) terms were used: ctDNA (MeSH) OR circulating tumor DNA OR liquid biopsy AND breast cancer (MeSH) OR breast carcinoma OR breast tumor AND prognosis (MeSH) OR survival. All titles were initially screened, and appropriate abstracts were reviewed. Each of the publications’ bibliographies and Google Scholar (Alphabet Inc) were manually searched for relevant articles. Where data in publications were insufficient, the authors contacted the authors of the relevant studies seeking permission to access data to extrapolate hazard ratios (HRs).

### Outcomes

The primary outcome was to determine the association of ctDNA with DFS or relapse-free survival in breast cancer. For the purpose of data reporting, DFS will include relapse-free survival and progression-free survival. Subgroup analysis was performed to identify the clinical utility of ctDNA in the metastatic vs early disease population. Similarly, subgroup analysis was performed to determine whether pretreatment or posttreatment sampling of ctDNA was associated with DFS. The quality of the studies included was assessed using the Newcastle-Ottawa Scale. For the Newcastle-Ottawa Scale, points were awarded for patient selection (maximum 4 points), outcome assessment (maximum 3 points), and comparability of cohort (maximum 2 points), for a maximum of 9 points.^16^ The risk of bias was assessed using the Risk of Bias in Non Randomized Studies of Intervention (ROBINS-I Tool; Cochrane Bias Methods Group).^17^ Publication bias was not assessed, as the number of studies was insufficient.

### Study Selection

The studies had to meet the following prespecified inclusion criteria: (1) a ctDNA blood sample was measured; (2) DFS, progression-free survival, or relapse-free survival was reported as an HR; and (3) the patient population only had breast cancer. Studies were excluded if cell-free DNA was measured, the outcomes of interest (DFS, relapse-free survival, or overall survival) were not reported, or the HR could not be extrapolated from the available data.

### Data Extraction

Two reviewers (C.C. and C.F.) independently reviewed the available literature according to the previously mentioned predefined strategy and criteria. The following variables were extracted: title and study details (year, journal, design, country), study population characteristics (sample size, target gene variant, cancer stage, method of quantification, time of sampling, outcome measure), and ctDNA data.

### Statistical Analysis

Statistical analysis was performed from July 30, 2019, to October 31, 2019, using Review Manager (RevMan), version 5 (The Cochrane Collaboration). Outcome data were reported as HRs. Any HRs reported in the study were used when available; otherwise, they were extrapolated using the available data. The 95% CIs were estimated using the Mantel-Haenszel method. An HR greater than 1 favored a worse outcome, ie, a reduced DFS or progression-free survival associated with elevated ctDNA levels. The 95% CI could not cross unity to be considered statistically significant. Heterogeneity was assessed using *I*^2^ statistics, with greater than 50% considered as significant heterogeneity. A fixed-effects model was preferred to a random-effects model when there was no significant heterogeneity and vice versa when there was significant heterogeneity (*I*^2^ > 50%). Pooled effect estimates (HRs) of differences were calculated using random-effects models, accounting for potential interstudy heterogeneity. All reported *P* values were two-sided, and *P* < .05 was considered statistically significant.

## Results

### Eligible Studies

A total of 263 publications were found using the predefined search terms, of which 8 studies (3.0%) were eligible for inclusion. Five (62.5%) prospective population studies were included,^18,19,20,21,22^ whereas the remaining 3 (37.5%) studies were retrospective cohort studies.^11,23,24^ All studies were published between 2002 and 2020. All studies were comparable, reporting the association between ctDNA levels and breast cancer outcomes. There was 100% agreement between the 2 reviewers on review of the extracted data. Study characteristics and Newcastle-Ottawa scores are presented in the Table.

**Table.** Study Characteristics and Newcastle-Ottawa Scores

Source	Country	Sample size	Target mutation	Quantification of ctDNA	Method of ctDNA analysis	Breast cancer subtype	Breast cancer stage	NOS	Pre or post Rx	Design	Outcomes
Coombes et al^20^	United Kingdom	49	Single nucleotide variants	Somatic variant detection	PCR	All	I-II	8	Post	Prospective	RFS
Ma et al^23^	China	37	Single nucleotide variants, *TP53*, *PIK3CA*, *MTOR*	Mutation cluster (heterogeneity)	PCR	All	I-III	8	Pre	Retrospective	PFS
Garcia-Murillas et al^18^	United Kingdom	101	*PIK3CA*	ctDNA detection	PCR	All	I-II	8	Pre	Prospective	RFS
Hu et al^11^	China	68	*TP53*, *PIK3CA*, *ERBB2*, *CDK12*	ROC curve for ctDNA levels	NGS	All	III/IV	8	Pre	Retrospective	PFS
Chen et al^19^	United States	38	*TP53*, *PIK3CA*, *AKT1*	Somatic mutation detection	NGS	TNBC	I-III	7	Post	Prospective	DFS
Chandarlapaty et al^21^	United States	156	*ESR1*	Mutation amplitude threshold	PCR	ER positive	IV	7	Post	Prospective	DFS
Fiegl et al^24^	Austria	148	Methylated *RASSF1A* DNA	*RASSF1A* methylation detection	DNA isolation	ER positive	I-II	6	Pre	Retrospective	DFS
Silva et al^22^	Spain	142	*TP53*	Loss of heterogeneity in polymorphic marker, mutation detection	PCR	Not specified	I-II	7	Pre	Prospective	PFS

Abbreviations: ctDNA, circulating tumor DNA; DFS, disease-free survival; ER, estrogen receptor; NGS, next-generation sequencing; NOS, Newcastle-Ottawa Scale; PCR, polymerase chain reaction; PFS, progression-free survival; RFS, relapse-free survival; ROC, receiver under the operating curve; Rx, diagnosis; TNBC, triple-negative breast cancer.

The studies differed in their methodology. Three studies examined ctDNA levels as a marker of progression-free survival among patients with locally advanced or metastatic breast cancer.^11,21,23^ Locally advanced disease refers to patients with axillary lymph node–positive disease. The other 5 studies reported ctDNA levels and DFS for patients with early breast cancer.^18,19,20,22,24^ The timing of ctDNA sampling also varied between studies. Five studies collected ctDNA samples before starting treatment (surgical or oncologic),^11,18,22,23,24^ whereas 3 studies recorded ctDNA after commencing treatment to predict oncologic outcomes.^19,20,21^

Quantification and measurement of ctDNA levels varied between the studies. Hu et al,^11^ Ma et al,^23^ and Chandarlapaty et al^21^ analyzed serial measurements of ctDNA in patients with metastatic breast cancer. Hu and colleagues^11^ used a receiver under the operating curve to determine the optimum ctDNA cutoff value for predicting disease progression. Ma et al^23^ and Chandarlapaty et al^21^ measured gene variation clusters and thresholds, respectively, to elicit the value of ctDNA variations. The remaining 5 studies examined early breast cancer disease and reported a delta trend in ctDNA over time.^18,19,20,22,24^ Circulating tumor DNA gene variation detection was reported as a poor prognostic indicator of DFS.

The target gene variations differed between studies. The most common genes that were targeted were *TP53* and *PIK3CA*. Hu et al^11^ measured *TP53*, *PIK3CA*, *ERBB2*, and *CDK12*. Similarly, Ma et al^23^ and Chen et al^19^ examined both the *TP53* and *PIK3CA* gene variations as well as *MTOR* and *AKT1*, respectively. Garcia-Murillas et al^18^ analyzed *PIK3CA* exclusively, whereas Silva et al^22^ studied the *TP53* mutation. The remaining 3 studies examined *ESR1*, *RASSF1A*, and single nucleotide variants, respectively.^20,21,24^ The follow-up time ranged from 12 months to 3 years.

### Patient Characteristics

A total of 739 patients were analyzed. Patient characteristics are reported in the Table. Hu et al,^11^ Ma et al,^23^ Garcia-Murillas et al,^18^ and Coombes et al^20^ examined all breast cancer subtypes, whereas Chen et al^19^ focused on triple-negative breast cancers. Estrogen receptor–positive breast cancer was the subtype studied by Fiegl et al^24^ and Chandarlapaty et al.^21^ Silva et al^22^ did not comment on the hormone profile of the breast cancers included in their study. Hu et al,^11^ Ma et al,^23^ and Chandarlapaty et al^21^ studied patients with locally advanced or metastatic disease. The remaining 5 studies examined early-stage breast cancer.^18,19,20,22,24^

### ctDNA Level and DFS

**Figure 1.**
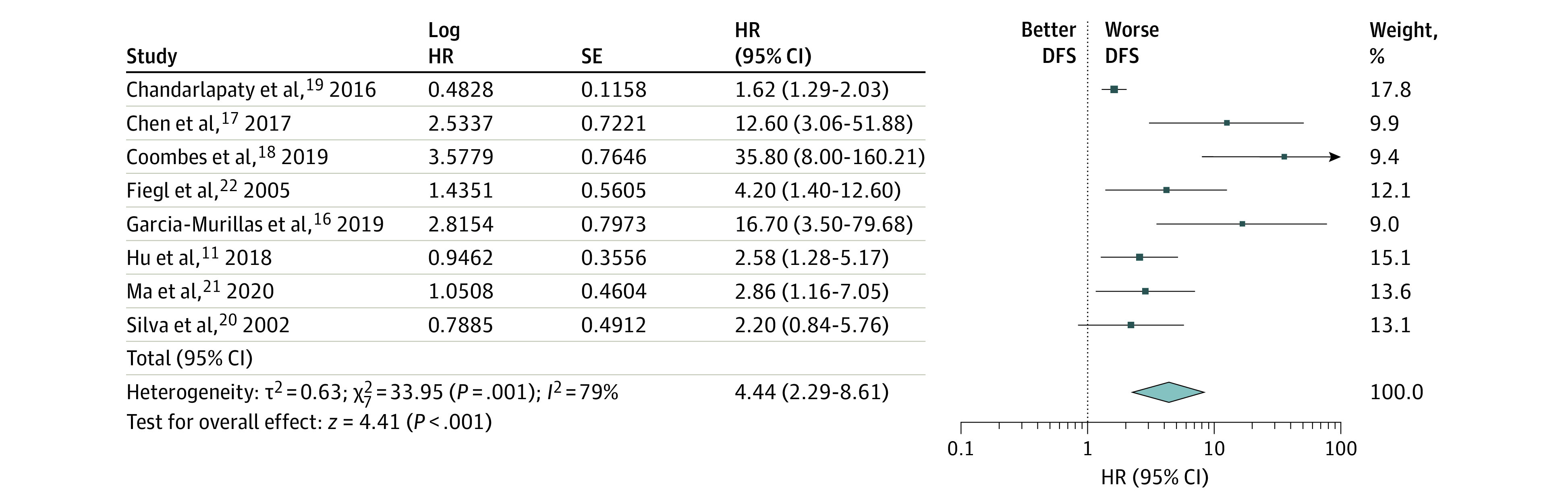
Association Between Elevated Circulating Tumor DNA Levels and Reduced DFS DFS indicates disease-free survival; HR, hazard ratio.

All 8 studies were included in the initial meta-analysis. There was a statistically significantly shorter DFS observed in patients with an elevated ctDNA level (HR, 4.44; 95% CI, 2.29-8.61; *P* < .001). There was statistically significant heterogeneity between these studies (*I*^2^ = 79%) (Figure 1).

**Figure 2.**
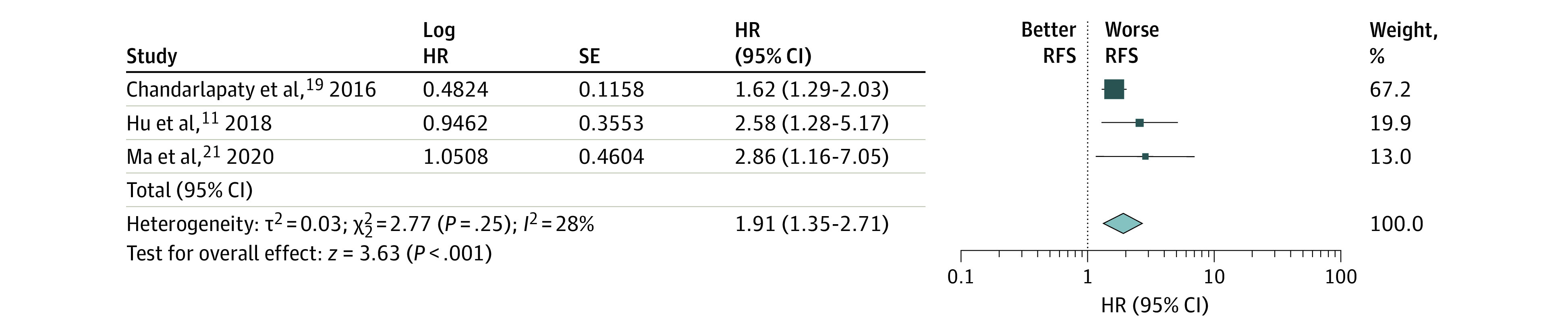
Subgroup Analysis of Metastatic or Locally Advanced Breast Cancer Group and Circulating Tumor DNA Levels HR indicates hazard ratio; RFS, relapse-free survival.

**Figure 3.**
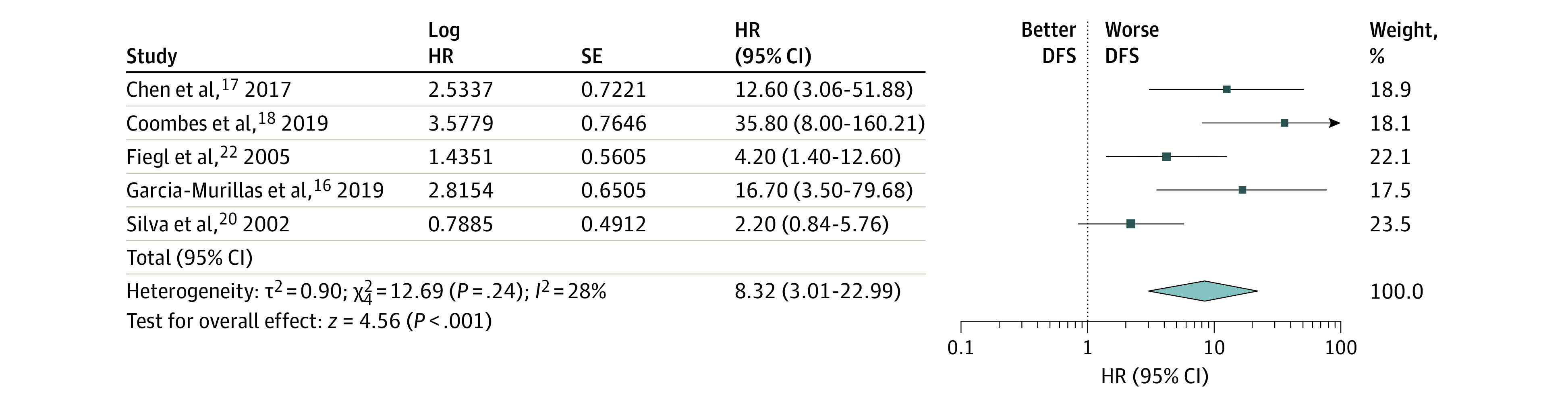
Subgroup Analysis of Early-Stage Breast Cancer Group DFS indicates disease-free survival; HR, hazard ratio.

Subgroup analysis was performed on the metastatic and locally advanced breast cancer patient cohort, and 3 studies were eligible for inclusion. Circulating tumor DNA level was statistically significantly associated with a lower relapse-free survival rate (HR, 1.91; 95% CI, 1.35-2.71; *P* < .001) with little heterogeneity (*I*^2 = ^28%) (Figure 2). With regard to the early breast cancer studies, a statistically significantly shorter DFS was associated with an elevated ctDNA level (HR, 8.32; 95% CI, 3.01-22.99; *P* < .001). However, there was no considerable heterogeneity between the studies (*I*^2^ = 28%) (Figure 3).

**Figure 4.**
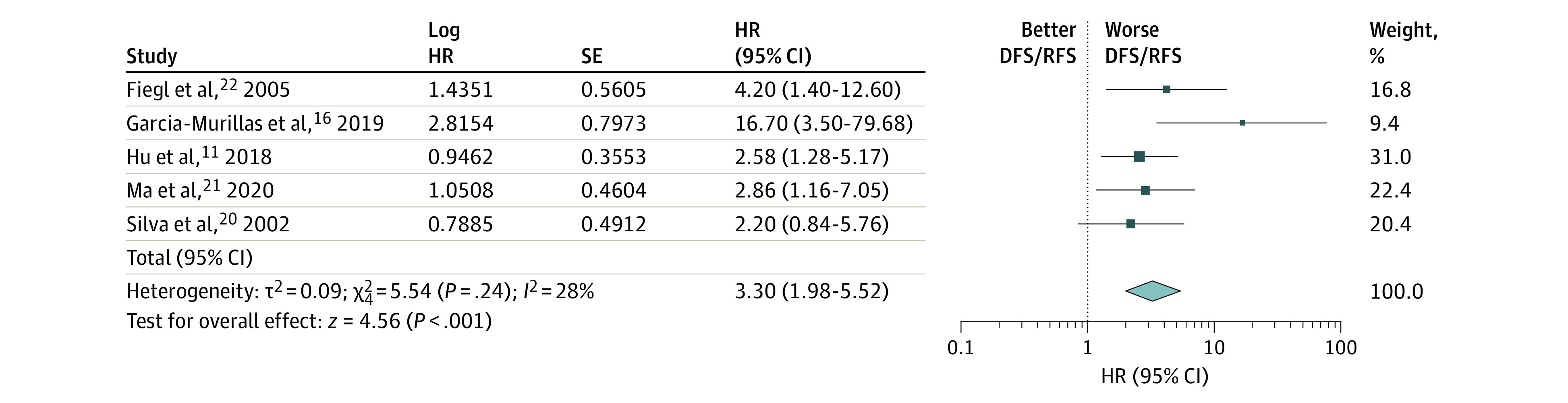
Subgroup Analysis of Pretreatment Sampling DFS indicates disease-free survival; HR, hazard ratio; and RFS, relapse-free survival.

To determine whether timing of ctDNA sample collection influenced the predictive value of ctDNA, a subgroup analysis was performed comparing pretreatment and posttreatment samples. Samples of ctDNA collected before commencement of oncologic or surgical treatment showed that patients with elevated ctDNA had a shorter DFS (HR, 3.30; 95% CI, 1.98-5.52; *P* < .001) with little heterogeneity (*I*^2^ = 28%) (Figure 4). Samples obtained after commencing oncologic treatment suggest a shorter DFS with elevated ctDNA levels (HR, 8.17; 95% CI, 1.91-65.89; *P* < .05). However, this outcome was statistically insignificant within a very heterogenous study group (*I*^2^ = 91%) (eFigure in the Supplement).

## Discussion

In this systematic review and meta-analysis of the published data, elevated ctDNA levels were associated with poorer cancer outcomes in patients with early, locally advanced, and metastatic disease. Subgroup analysis from this study found that ctDNA gene variation detection before the commencement of treatment was associated with unfavorable survival outcomes. This finding refutes the potential for surgical insult to be the source of elevated ctDNA levels. To the best of our knowledge, this is the first meta-analysis solely examining the association of ctDNA gene variation status and breast cancer outcomes.

These findings concur with a previously published meta-analysis reporting on the utility of liquid biopsy in breast cancer.^25,26^ Lee et al^26^ analyzed 69 studies to determine the prognostic value of liquid biopsy in predicting lymph node metastases, recurrence, and survival in breast cancer. Similarly, Tan et al^25^ reported that circulating cell-free DNA is a strong predictive and prognostic marker in patients with breast cancer. Both of these meta-analyses included studies with cell-free DNA, ctDNA, and circulating tumor cells.

Circulating tumor DNA as a predictive biomarker is gaining much appreciation in the field of colorectal cancer, pancreatic cancer, and gastric cancer, among many others.^27,28,29,30^ Circulating tumor DNA was first discovered in 1948; however, it has only recently come to prominence because of advances in technologic development and detection methods.^31^ Circulating tumor DNA is composed of tiny fragments, accounting for as little as 0.01% of the total circulating DNA (cell-free DNA) with a very short half-life (less than 2 hours).^32^ One of the original methods for detecting ctDNA involved DNA isolation, bisulfite modifications, and MethyLight analysis;^31,33^ this was the detection method adopted by Fiegl et al^24^ in 2005.

In more recent years, sophisticated polymerase chain reaction (PCR)–based techniques have been adopted to detect ctDNA in various cancers. One of the practical limitations of PCR is that knowledge of the particular genetic variation is required before it can be quantified. Tumor analysis is necessary before gene variation alteration measurement unless the target is well established, such as *TP53*, *PIK3CA*, and *ERBB2* in breast cancer.^34^ Five of the studies included in the present study used PCR techniques to measure ctDNA gene variants.^18,20,21,22,23^ Detection of ctDNA by next-generation sequencing (NGS)–based techniques is an advancement in tumor prognostics, as this technique sequences millions of DNA templates in parallel, enabling rapid identification of tumor-specific alterations in ctDNA.^35^ Unlike with PCR-based techniques, prior knowledge of the underlying gene variation is not required in order to quantify genomic alterations using NGS. Furthermore, NGS-based techniques allow for detection of several variations in multiple genes. Hu et al^11^ and Chen et al^19^ used NGS-based techniques to collate data for their studies. Chen et al^19^ included patients with early-stage disease, suggesting that NGS is a sensitive detection platform in all stages of disease. Recent advances in PCR- and NGS-based techniques have paved the way for ctDNA as a potential prognostic biomarker in breast cancer.

The clinical application of ctDNA detection in breast cancer lies in its prognostic potential. With the advent of Oncotype DX (Genomic Health Inc) and MammaPrint (Agendia Inc), the application of chemotherapy is becoming more individualized, and overtreatment should be avoided if possible. Oncotype DX is a clinically validated 21-gene genomic assay that can quantify the risk of breast cancer recurrence and the degree of benefit associated with chemotherapy.^35^ This tool has revolutionized oncologic management of patients with early-stage breast cancer; however, the score does not absolutely reflect residual disease burden. There is a need for circulating biomarkers that can reflect tumor burden and highlight selected patients who would benefit from adjuvant systemic treatment. The present meta-analysis shows that ctDNA can reflect tumor burden and be used to monitor response to therapy.

The clinical utility of ctDNA as a prognostic marker can be applied to both early and metastatic breast cancer disease as suggested by subgroup analysis in this study. Another potential benefit of ctDNA is that it can detect residual disease after neoadjuvant therapy. McDonald et al^36^ demonstrated high accuracy for assessment of molecular response and residual disease during neoadjuvant therapy using ctDNA analysis. That study was limited by the fact that 1 patient had high-volume residual disease post–neoadjuvant therapy with undetectable ctDNA, potentially due to a limited number of gene variants assayed for this patient. The usefulness of ctDNA as a prognostic biomarker during neoadjuvant therapy has been validated by a number of further studies with various breast cancer subtypes.^37,38^ In the context of metastatic breast cancer and multiline chemotherapy resistance, Hu et al^11^ proposed that drug selection should be streamlined by identifying drug-sensitive gene variations in ctDNA.

Despite advances in surgical and oncologic treatment, breast cancer recurrence rates remain significant. The hazard of recurrence is highest during the first 5 years after initial treatment (10.4%), with a peak during the first 2 years.^39^ Current surveillance methods used to detect asymptomatic patients are based on clinical examination and radiologic imaging. Garcia-Murillas et al^18^ performed a multicenter prospective study on a cohort of 101 female patients and found that ctDNA analysis detected disease relapse before it became radiologically or clinically apparent with a median time of 10.7 months. Brain-only metastasis was less commonly detected by ctDNA, which is an important consideration should ctDNA analysis be incorporated in follow-up surveillance.^18^

### Limitations

There are a number of limitations to this meta-analysis. The main limitation is the heterogeneity of the included studies, which is reflected in the wide CIs. A random-effects model was adopted in an attempt to account for significant interstudy heterogeneity. Another drawback is that all of the relevant literature pertaining to this subject could not be included in the analysis owing to lack of data available. Efforts were made to obtain relevant data from the different authors, but correspondence was not always reciprocated. Regarding the aforementioned methods for ctDNA analysis, the included studies differed in the techniques they adopted for quantifying ctDNA. A standardized technique needs to be established in order to introduce ctDNA analysis into routine clinical practice.

## Conclusions

In conclusion, this meta-analysis found that ctDNA detection was associated with decreased DFS and progression-free survival in patients with breast cancer. The clinical utility of ctDNA ranged from early breast cancer cohorts to patients with metastatic breast cancer with multiline drug resistance (the latter with the tightest CI). The use of ctDNA as a clinical biomarker has the potential to identify preclinical disease recurrence in patients after breast cancer treatment. In an era of personalized medicine, ctDNA shows promise as a tool for guiding future precision medicine.

## Notes

Supplement. **eFigure.** Subgroup Analysis of Post-Treatment Sampling. Elevated ctDNA Levels Associated With Worse DFS/RFS (HR 8.17, 95% CI 1.01-65.89, *P* > .05)
