# Supplementary material for: Association of Circulating Tumor DNA With Disease-Free Survival in Breast Cancer: A Systematic Review and Meta-analysis
Source: JAMA Netw Open. 2020 Nov 19;3(11):e2026921. doi: 10.1001/jamanetworkopen.2020.26921 (PMC7677763; doi:10.1001/jamanetworkopen.2020.26921)
Supplement: Supplement. — eFigure. Subgroup Analysis of Post-Treatment Sampling. Elevated ctDNA Levels Associated With Worse DFS/RFS (HR 8.17, 95% CI 1.01-65.89, P > .05) [file jamanetwopen-e2026921-s001.pdf]

Supplementary Online Content

Cullinane C, Fleming C, O'Leary DP, et al. Association of circulating tumor DNA with disease-free survival in breast cancer: a systematic review and meta-analysis. *JAMA Netw Open*. 2020;3(11):e2026921. doi:10.1001/jamanetworkopen.2020.26921

eFigure. Subgroup Analysis of Post-Treatment Sampling. Elevated ctDNA Levels Associated With Worse DFS/RFS (HR 8.17, 95% CI 1.01-65.89, P > .05)

This supplementary material has been provided by the authors to give readers additional information about their work.

© 2020 Cullinane C et al. *JAMA Network Open.*

eFigure. Subgroup Analysis of Post-Treatment Sampling. Elevated ctDNA Levels Associated With Worse DFS/RFS (HR 8.17, 95% CI 1.01-65.89, P > .05)

Study or Subgroup	log[Hazard Ratio]	SE	Weight	Hazard Ratio IV, Random, 95% CI
Chandarlapaty 2016	0.4824	0.1158	37.0%	1.62 [1.29, 2.03]
Chen 2017	2.5337	0.7221	31.8%	12.60 [3.06, 51.88]
Coombes 2019	3.5779	0.7646	31.2%	35.80 [8.00, 160.21]
**Total (95% CI)**			**100.0%**	**8.17 [1.01, 65.89]**

Heterogeneity: $Tau^2 = 3.05$; $Chi^2 = 23.37$, df = 2 (P < 0.00001); $I^2 = 91\%$
Test for overall effect: Z = 1.97 (P = 0.05)

Hazard Ratio
IV, Random, 95% CI

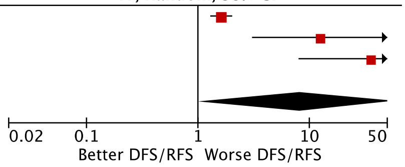

© 2020 Cullinane C et al. *JAMA Network Open.*
